# A Patient Decision Aid (i.ARTs) to Facilitate Women’s Choice Between Oral and Long-Acting Injectable Antiretroviral Treatment for HIV: Protocols for its Development and Randomized Controlled Pilot Trial

**DOI:** 10.2196/35646

**Published:** 2022-09-13

**Authors:** Morgan M Philbin, Tara McCrimmon, Victoria A Shaffer, Deanna Kerrigan, Margaret Pereyra, Mardge H Cohen, Oluwakemi Sosanya, Anandi N Sheth, Adaora A Adimora, Elizabeth F Topper, Aadia Rana, Bani Tamraz, Lakshmi Goparaju, Tracey E Wilson, Maria Alcaide

**Affiliations:** 1 Department of Sociomedical Sciences Columbia University Mailman School of Public Health New York, NY United States; 2 Department of Health Sciences University of Missouri Columbia, MO United States; 3 Department of Prevention and Community Health Milken Institute School of Public Health George Washington University Washington, DC United States; 4 Department of Medicine John H. Stroger Jr Hospital of Cook County Chicago, IL United States; 5 Department of Medicine Montefiore Medical Center Bronx, NY United States; 6 Department of Medicine Emory University School of Medicine Atlanta, GA United States; 7 Department of Medicine Department of Epidemiology University of North Carolina Chapel Hill Chapel Hill, NC United States; 8 Department of Epidemiology Johns Hopkins Bloomberg School of Public Health Baltimore, MD United States; 9 Department of Medicine University of Alabama at Birmingham School of Medicine Birmingham, AL United States; 10 School of Pharmacy University of California San Francisco San Francisco, CA United States; 11 Department of Medicine Georgetown University Medical Center Washington, DC United States; 12 Department of Community Health Sciences State University of New York Downstate Health Sciences University Brooklyn, NY United States; 13 Department of Medicine University of Miami Miller School of Medicine Miami, FL United States

**Keywords:** patient decision aid, HIV treatment, oral ART, long-acting injectable ART, study protocol, women’s health

## Abstract

**Background:**

Many women with HIV (WWH) have suboptimal adherence to oral antiretroviral therapy (ART) due to multilevel barriers to HIV care access and retention. A long-acting injectable (LAI) version of ART was approved by the US Food and Drug Administration in January 2021 and has the potential to overcome many of these barriers by eliminating the need for daily pill taking. However, it may not be optimal for all WWH. It is critical to develop tools that facilitate patient-provider shared decision making about oral versus LAI ART modalities to promote women’s adherence and long-term HIV outcomes.

**Objective:**

This study will develop and pilot test a web-based patient decision aid called i.ART+support (i.ARTs). This decision aid aims to support shared decision making between WWH and their providers, and help women choose between oral and LAI HIV treatment.

**Methods:**

The study will occur in 3 phases. In phase 1, we will utilize a mixed methods approach to collect data from WWH and medical and social service providers to inform *i.ARTs* content. During phase 2, we will conduct focus groups with WWH and providers to refine *i.ARTs* content and develop the web-based decision aid. In phase 3, *i.ARTs* will be tested in a randomized controlled trial with 180 women in Miami, Florida, and assessed for feasibility, usability, and acceptability, as well as to evaluate the associations between receiving i.ARTs and viral suppression, ART pharmacy refills, and clinic attendance.

**Results:**

This study was funded in March 2021. Columbia University’s IRB approved the study protocols (approval number IRB-AAAT5314). Protocols for phase 1 interviews have been developed and interviews with service providers started in September 2021. We will apply for Clinicaltrials.gov registration prior to phase 3, which is when our first participant will be enrolled in the randomized controlled trial. This is anticipated to occur in April 2023.

**Conclusions:**

This study is the first to develop a web-based patient decision aid to support WWH choices between oral and LAI ART. Its strengths include the incorporation of both patient and provider perspectives, a mixed methods design, and implementation in a real-world clinical setting.

**International Registered Report Identifier (IRRID):**

DERR1-10.2196/35646

## Introduction

Suboptimal antiretroviral therapy (ART) adherence among people with HIV has constrained efforts to curb the HIV epidemic in the United States [1]. Women face myriad barriers to HIV care and treatment and have historically been underrepresented in clinical trials for HIV treatment [2,3]. As a result, women with HIV (WWH) have lower care retention (58% retention versus 65% retention in the overall US population), which contributes to their increased mortality compared with men [1,4-7]. There is therefore an urgent need for strategies that optimize care engagement and viral suppression among WWH.

Long-acting injectable (LAI) ART may be a strategy to improve ART adherence and HIV outcomes for women [2,3,8]. The first LAI ART (cabotegravir/rilpivirine), which consists of monthly intramuscular injections rather than daily pills, was approved by the US Food and Drug Administration (FDA) in January 2021; a bimonthly version was approved in February 2022; and other LAI ART drugs are in advanced stages of clinical trials and expected to be available for HIV treatment in the near future [9]. Women comprised only 8%-33% of phase 2 and phase 3 LAI ART trial participants, and pregnant women were not included [10-13]. As such, gender-specific barriers, pregnancy-related interactions [13] and characteristics that promote ART adherence among WWH remain underexplored [14-16]. Furthermore, LAI ART research has occurred largely among clinical trial participants, whose optimal medication adherence and clinic attendance do not represent the majority of WWH [17].

While preliminary research suggests that most WWH would prefer LAI ART over their current daily oral medication [18], this research has also identified multilevel barriers to LAI ART uptake. At the *individual level*, these include women’s concerns about side effects, pregnancy-related interactions, and drug resistance if LAI ART is discontinued without oral ART initiation; at the *clinic level*, barriers include medical mistrust due to historic sterilization campaigns [19] and lack of provider knowledge and willingness to offer LAI ART; and at the *structural level*, barriers include gender-specific socioeconomic inequalities and dynamics such as low-wage employment with unstable scheduling, lack of transportation, and care-taking responsibilities. Furthermore, FDA indication still requires viral suppression via oral ART prior to initiating LAI. These multilevel barriers could all complicate the frequent visits that LAI ART administration will require [18,20-23]. WWH and providers may benefit from tools and strategies that help them to identify and address barriers that prevent uptake of, and adherence to, LAI ART.

Shared decision making in medical settings is often accomplished using patient decision aids. These evidence-based tools promote equity in medicine by increasing patients’ knowledge, decision-making power [24,25], and health outcomes [26-28]. This approach can also lower medical paternalism [24,29-31]. Patient decision aids can improve medication adherence directly [32], as well as through mediating factors (eg, patient satisfaction [33-35], efficacy [36-38], and communication) [33,39,40]. These tools are ideal for preference-sensitive decisions with multiple options [41,42]; however, no patient decision aids yet exist to facilitate women’s choice between HIV treatment modalities. New tools are urgently needed to ensure the successful and equitable integration of new technologies such as LAI ART into clinical settings [43].

This paper describes the protocol for a mixed methods study to develop, refine, and test a patient decision aid for WWH, called i.ART+support (*i.ARTs*). *i.ARTs* will facilitate shared decision making between WWH and providers to determine which HIV treatment (oral or LAI ART) best fits a woman’s preferences, addresses her unique barriers, and, in doing so, facilitates adherence. This study will be conducted within the MACS/WIHS Combined Cohort Study (MWCCS) [44] and will fill the aforementioned gaps in existing research regarding patient decision aids for WWH. MWCCS is the largest and oldest prospective epidemiological cohort study of HIV in the United States. The current cohort includes approximately 1800 women from geographically diverse sites across the United States. Each of these has associated clinical sites with medical and social service providers who work with WWH. The aim of this paper is to provide an overview of all procedures for our study focused on developing a patient decision aid to support WWH as they choose between LAI and oral formulations of ART.

## Methods

### Overview of the Study

**Figure 1:**
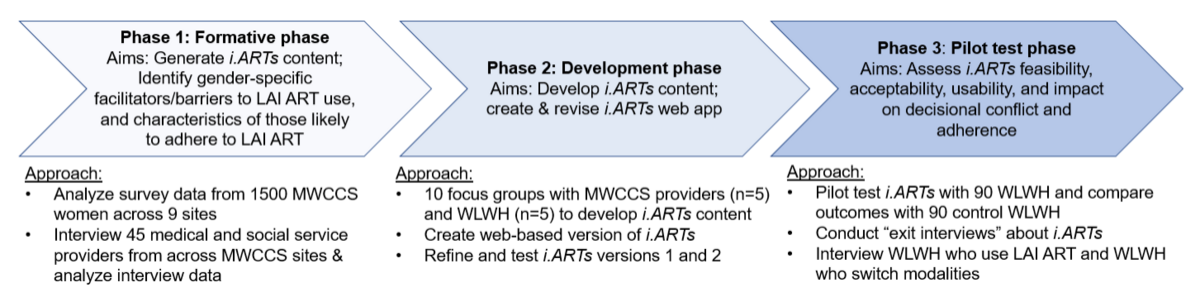
Study overview. *i.ARTs*: i.ART+support; LAI ART: long-acting injectable ART; MWCCS: MACS/WIHS Combined Cohort Study; WWH: women with HIV.

Study methods are aligned with current systematic approaches to patient decision aid development [36,45,46], and will occur in 3 phases (Figure 1). Each phase is associated with 1 of the following study aims. *Phase 1* will generate data to inform *i.ARTs* content using mixed methods research with WWH and providers. *Phase 2* will iteratively develop *i.ARTs* as a web-based patient decision aid. *Phase 3* will pilot test *i.ARTs* to assess feasibility, acceptability, and usability and to compare decisional outcomes and adherence data (including viral suppression, ART refills, and clinic attendance) between *i.ARTs* recipients and standard of care (control) WWH (n=180; 90 per group). Each phase includes distinct data collection activities, which are summarized in Table 1.

**Table 1:** Overview of data sources and participants.

Phase	Data source	Participants	Scope of data	Purpose
1	MWCCS^a^ 2020 cohort survey	1500 WWH^b^ at 9 MWCCS sites	WWH perspectives on LAI ART^c^ and decision making; identify relevant barriers and facilitators to LAI ART uptake; characteristics of women who are most likely to adhere to LAI ART	Generate *i.ARTs*^d^ content
1	Provider interviews	45 medical/social service providers at MWCCS sites	Provider perspective on characteristics of women most likely to adhere to LAI ART; reasons providers would not offer LAI ART to a woman for whom it is clinically indicated; provider perceptions of multilevel barriers and facilitators to WWH’s ART use	Generate *i.ARTs* content
2	Focus groups	MWCCS providers, WWH in Miami, Florida	Feedback on successive iterations of *i.ARTs*	Develop and refine *i.ARTs* content
3	Baseline and postvisit survey	180 WWH (90 from the intervention/*i.ARTs* arm and 90 from the control arm)	Information on decisional conflict (baseline and post), acceptability and satisfaction (post only), and sociodemographic and behavioral moderators (baseline only)	Assess *i.ARTs* acceptability and usability outcomes, assess impact of moderators
3	Exit interviews	20 WWH who receive *i.ARTs* and 10 providers	WWH and provider perspectives on *i.ARTs* tool	Assess *i.ARTs* feasibility, usability, acceptability
3	Electronic medical record data	180 WWH	*i.ARTs* adherence data for study participants, including clinic visits, medication refills, and viral load data	Preliminary impact of *i.ARTs* data on treatment-related outcomes

^a^MWCCS: MACS/WIHS Combined Cohort Study.

^b^WWH: women with HIV.

^c^LAI ART: long-acting injectable antiretroviral therapy.

^d^*i.ARTs*: i.ART+support

### Phase 1: Formative Work to Inform *i.ARTs* Content

To ensure *i.ARTs* addresses a comprehensive array of factors that influence LAI ART uptake, phase 1 includes data collection that incorporates the perspectives of both WWH and providers at MWCCS sites.

*Phase 1a* will quantitatively assess WWH perspectives on LAI ART and decision making, identify relevant barriers and facilitators to LAI ART uptake, and identify the characteristics of women who are most likely to adhere to LAI ART. It utilizes survey data administered to the MWCCS study cohort starting in October 2020. Historically, this sample included 1661 WWH of whom 1610 have a history of taking oral ART [44]. None have participated in LAI ART clinical trials. Full descriptions of participant recruitment, selection, enrollment, and study procedures for MWCCS are available elsewhere [44]. Survey responses from the 2020 cohort were collected from fall 2020 through summer 2021, and include 15 items that assess LAI ART–related knowledge, interest, and potential barriers and facilitators to use. We will use multivariable logistic regression models to identify factors associated with WWH’s interest in, and barriers and facilitators to, LAI ART. While potential barriers and facilitators to LAI ART uptake can change over time (eg, transportation/housing), we will be looking at these associations cross sectionally and thus capturing these barriers at a given moment in time. The sample size provides the power to detect modest effect sizes in a multivariable logistic regression analysis (odds ratios ranging from 1.4 to 1.6), even with a low probability of LAI interest and a high squared multiple correlation (0.4-0.5) between predictor variables. This sample size will allow for subgroup analyses and still detect moderate effect sizes (eg, by age or MWCCS site).

*Phase 1b* assesses HIV provider perspectives on LAI ART for their female clients. It involves in-depth interviews with 30 medical (eg, infectious disease physicians, advanced practice registered nurses) and 15 social service providers (eg, case managers, HIV clinical social workers) who serve WWH across 9 MWCCS sites and affiliated clinical sites.

Provider participants will complete a 1-time, 45-60-minute interview, and receive a US $75 gift card as compensation. Interview domains will include (1) providers’ perceived characteristics of women most likely to adhere to LAI ART, particularly compared with oral ART (eg, housing, oral ART adherence, caregiving responsibilities, and clinic attendance); (2) reasons providers would not offer LAI ART to a woman for whom it is clinically indicated; (3) providers’ perspectives on decision making in the clinical setting and their experience in deciding which ART medications their WWH clients should take; and (4) multilevel barriers and facilitators to WWH’s ART use and solutions to overcome these adherence barriers. These domains are determined by formative work by the study team [18,20,22,23]. Interviews will be digitally recorded and professionally transcribed. Transcripts will be coded using a set of codes inductively identified from the data; these will be supplemented by codes derived from the existing literature. A thematic content analysis approach will be used to identify key findings within domains of interest [47]. Data will be summarized and applied to activities in phase 2, described in the following section.

### Phase 2: *i.ARTs* Development

Phase 2 utilizes sequential focus groups with both WWH and medical and social service providers to determine the content of *i*.*ARTs* and create and revise the *i.ARTs* web-based app. Existing research indicates that web-based decision aid tools have advantages over paper-based ones, including interactive interfaces and visual filtering and sorting options [48]. This stage of *i.ARTs* development will follow International Patient Decision Aids Standards Collaboration (IPDAS) guidelines [46,49,50] and use the Ottawa Decision Support Framework [27,51-53]. This framework explains the relationship between participants’ decisional needs and decisional quality using both prescriptive [54] (based on rational actions, highest expected utility) and descriptive [55] (preferences-based, nonrational) decision theories.

**Figure 2:**
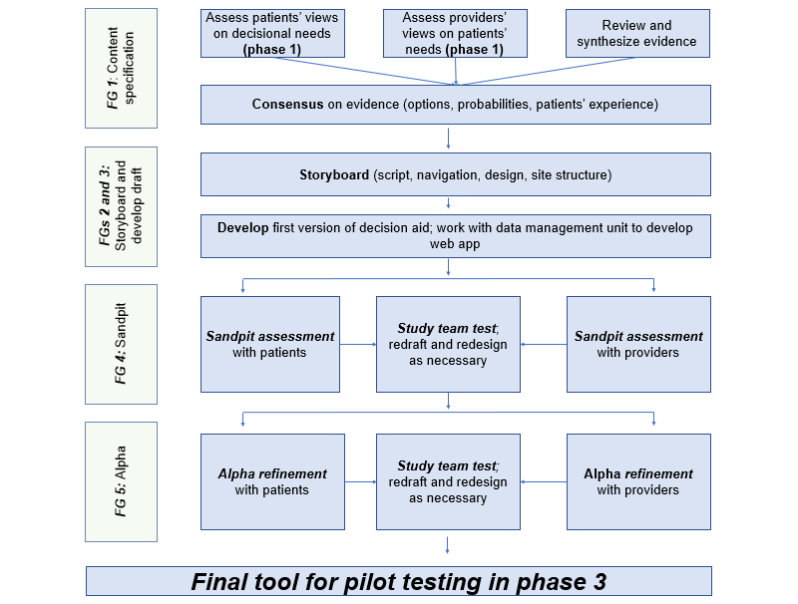
*i.ARTs* decision aid development process and focus groups (FGs). *i.ARTs*: i.ART+support.

An overview of the planned *i.ARTs* development process is included in Figure 2. We will conduct 2 sets of 5 focus groups: 1 set with 10 MWCCS providers recruited from the Phase 1 interview participants and the other set with 8-10 WWH recruited from the Miami MWCCS–affiliated clinical site. The Miami MWCCS site was selected due to the city’s high HIV incidence and low ART adherence, as well as a racially/ethnically diverse population [56-58]. WWH will be selected from Miami’s MWCCS community advisory board as well as by local MWCCS collaborators to ensure inclusion of women with a diverse range of perspectives, clinic attendance, and adherence. Each focus group will meet for approximately 60 minutes, and participants will be compensated US $40 per session for their time. We will record and transcribe the focus groups and review them to identify which items and topics should be included in the patient decision aid, as well as how to word specific items.

**Figure 3:**
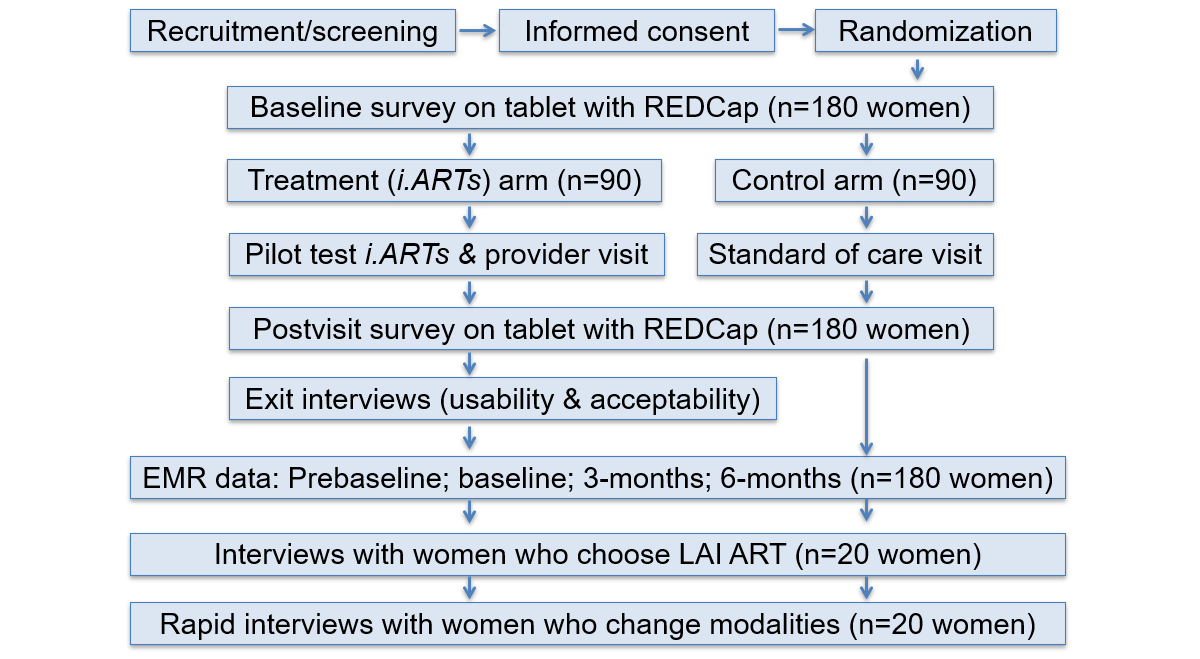
Pilot study flow and assessments. EMR: electronic medical record; *iARTs*: i.ART+support; LAI ART: long-acting injectable antiretroviral therapy.

Focus groups will use data from phase 1 to develop and finalize *i.ARTs* content and iteratively refine the web-based version of *i.ARTs* to prepare for the phase 3 pilot testing according to the schedule in Figure 3. Between each set of focus group meetings, the study team will integrate feedback from both the provider and WWH groups and either create the suggested content or make the suggested improvements to the *i.ARTs* program. This will ensure that women’s perspectives and realities will be captured to tailor the content within the patient decision aid.

### Phase 3: Pilot Test *i.ARTs*

#### Study Design

Phase 3 will pilot test the final *i.ARTs* patient decision aid to assess feasibility, acceptability, and usability as well as to evaluate associations between receipt of *i.ARTs* and ART adherence, including viral suppression, ART refills, chosen ART modality, and clinic attendance. The pilot will be a 2-arm randomized controlled trial with 180 WWH. It will compare an intervention arm (n=90 WWH) that uses the *i.ARTs* decision aid tool developed in phases 1 and 2 with a standard-of-care control arm (n=90 WWH).

#### Setting, Participants, Recruitment, and Enrollment

Pilot testing will occur among WWH at the same Miami MWCCS clinical site where phase 2 focus groups occurred. The clinic is one of the largest hospital-based clinics in Miami, a city with one of the highest rates of incident and prevalent HIV infections in the United States. We will recruit 180 women from the existing client population over the course of 15 months (about 12 women per month). A full-time research associate will approach potential participants at their regular clinic visit, share information about the study, and invite them to take part in eligibility screening. Participants must meet the following eligibility criteria: (1) identify as female; (2) be receiving HIV-related care at the Miami site; (3) have a diagnosis of HIV infection; (4) aged 18 and older; (5) be interested in learning about LAI ART and open to discussing HIV treatment with their provider; and (6) be willing to provide informed consent.

#### Study Procedures

##### Randomization

Following informed consent and the baseline assessment (discussed later), we will use a web-based system to randomize women (1:1) to the *i.ARTs* intervention arm (n=90) or to the standard-of-care control arm (n=90). Neither participants nor researchers will be blind to study condition. Women in the control arm will receive standard of care as it exists at the time of LAI ART roll out: providers will offer women information about LAI ART and may strongly suggest which option she should choose. Women who participated in the focus groups will not be eligible for inclusion.

Women in the intervention arm will use *i.ARTs* as part of their regular participant visit. While the complete content of *i.ARTs* will be finalized in phase 2, the web-based *i.ARTs* app will guide WWH through the following activities: (1) provide education on different ART modalities, (2) utilize value clarification methods to guide women in ranking the prominence of relevant barriers and facilitators that may impact adherence to various ART modalities, (3) collect information on participant health history, (4) generate a suggested ART modality based on previous steps, (5) assist WWH in developing questions for their health care providers, and (6) generate an individual profile description as well as individualized suggestions to support adherence based on the modality selected (eg, a woman may be profiled as having transportation challenges; *i.ARTs* would suggest solutions—arranged transportation or transportation vouchers—that the clinic could employ to address these challenges). Participants will take a printout of this profile to their clinic visit with their provider. Intervention arm participants will receive US $15 for *i.ARTs* use.

##### Assessments

All women enrolled in the study will complete a baseline survey following informed consent and a follow-up survey immediately after their clinical visit. Surveys will be administered on tablets in a private room within the clinic and will use REDCap for data collection. Each survey will take approximately 25 minutes to complete, and women will receive US $25. Survey measures are described later and in Table 2.

A subsample of 20 intervention (*i.ARTs*) arm women and 10 providers will be randomly chosen to participate in an exit interview. This interview will occur either at the baseline visit or up to 1 month after *i.ARTs* use at the participant’s convenience. We will select every fourth woman in the intervention arm to complete the exit interview. Providers will be selected based on the number of *i.ARTs* participants they served in the first year of the pilot (we will randomly select 10 providers from all providers who serve above the median number of *i.ARTs* clients, which ensures that providers will have sufficient experience with the tool to provide feedback) and exit interviews with providers will be conducted in the final 3 months of data collection. These interviews will focus on patient and provider experiences with *i.ARTs* acceptability, feasibility, and usability, and the most useful ways to improve its integration into clinical practice. This will also include discussions of how women shared the results from the patient decision aid with their provider. Exit interviews will take approximately 30 minutes and participants will be provided US $30 in compensation.

The study will also use all 180 participants’ electronic medical records (EMRs) to assess the impact of the intervention on clinical outcomes (Table 2). The informed consent process will include the EMR abstraction release form and detailed information about the data abstraction process. Study staff will abstract data on viral suppression (all women), ART modality, clinic attendance (all women), and medication refill data (women on oral ART) from EMRs into a REDCap database. Abstraction will include data from 1-year prebaseline to 6 months postbaseline. This timeline is based on the frequency of HIV clinic visits (ie, approximately every 3 months) to ensure that women have at least one visit in the follow-up period.

**Table 2:** *i.ARTs*^a^ pilot study measures in phase 3.

Outcomes			Description of measures
**Primary**			Assessed after *i.ARTs* use among women using i.ARTs (n=90)
	Feasibility	Adaptation of Thabane et al’s [59] framework [60,61] for assessing pilot studies: Process, Resources, Management, Science	
	Acceptability and usability	Acceptability [62] and CSQ-8^b^ [63] to measure satisfaction with *i.ARTs*; exit interviews about *i.ARTs* features/protocol [62]	
**Secondary**			Assessed in baseline survey and postvisit survey for all women (n=180)
	Decisional conflict	Decisional Conflict Scale (16 items) [52]; satisfaction with the chosen method [64]	
**Tertiary**			Assessed 1-year prebaseline, at baseline, 3 months, and 6 months (all women; n=180)
	Viral suppression	Viral suppression, defined as viral load below limit of detection per assay used	
	Medication refills	Missed doses; report of medication refills/pharmacy pick up (WWH^c^ on oral ART^d^)	
	Clinic attendance	Missed or cancelled medical visits (<2 visits in a 6-month period for oral ART; missed monthly visit for LAI^e^ ART)	
	ART modality	Adoption of LAI among patients who are virally suppressed	
**Moderators**			Assessed at baseline
	Sociodemographics	Race/ethnicity, age, housing stability, education, employment, income, distance from clinic, relationship status, children	
	Depression/anxiety	PHQ-8^f^ (depression) [65]; GAD-7^g^ [66]	
	Substance use	ASSIST^h^ [67], an 8-item measure of problematic substance use	
	Stigma/discrimination	MDS^i^ to measure racial discrimination [68]; HIV-related stigma	
	Self-efficacy	HIV-ASES^j^ [38]	
	ART-related knowledge	Adaptation of Feldman’s [69] scale of oral ART knowledge; BMQ^k^ [70]	

^a^*i.ARTs*: i.ART+support.

^b^CSQ-8: 8-item Client Satisfaction Questionnaire.

^c^WWH: women with HIV.

^d^ART: antiretroviral therapy.

^e^LAI: long-acting injectable.

^f^PHQ-8: 8-item Patient Health Questionnaire.

^g^GAD-7: 7-item General Anxiety Disorder scale.

^h^ASSIST: Alcohol, Smoking and Substance Involvement Screening Test.

^i^MDS: 10-item Multiple Discrimination Scale.

^j^HIV-ASES: HIV Treatment Adherence Self-Efficacy Scale.

^k^BMQ-18: 18-item Beliefs and Medicines Questionnaire.

#### Study Outcomes and Measures

All study outcomes are summarized in Table 2. *Primary outcomes* include *i.ARTs feasibility, acceptability,* and *usability.* To assess feasibility, we will adapt Thabane et al’s [59] framework [60,61]. For *acceptability and usability,* we will use the 8-item Client Satisfaction Questionnaire (CSQ-8) to assess participants’ attitudes, burden, ethics, coherence, opportunity cost, perceived effectiveness, and self-efficacy [63]. The *secondary outcome* is ART-related decisional conflict, a 16-item scale scored from 0 to 100 [52]. Scores of 0-25 are associated with “implementing decisions” and scores of 37.5-100 are associated with “decision delay/feeling unsure.” *Tertiary outcomes* are exploratory, and consist of information extracted from EMRs, including viral suppression at the most recent visit in the prior 6 months, ART modality, medication refills (oral ART), and clinic attendance in the prior 6 months.

In addition to the outcomes, the baseline survey will measure moderators associated with adherence, including sociodemographics, depression, anxiety, substance use, HIV-related stigma and discrimination, self-efficacy, and ART-related knowledge.

#### Analysis

The primary outcomes of feasibility, acceptability, and usability will be assessed descriptively. We will compare ART-related decisional conflict, clinic attendance, viral suppression, and medication refills between and within the 90 women in each arm (baseline vs postvisit surveys and EMR data at 3 and 6 months). For normally distributed data, we will first use *t* tests to compare the 2 arms and then use regression methods to develop a multivariable model of the outcome, with arm membership as the principal covariate, while controlling for the moderators listed in the previous sections and in Table 2. For nonnormally distributed data, we will employ chi-square tests and logistic regression; distributional properties of the outcome will determine the type of logistic regression (eg, binary, multinomial, or ordinal logistic regression models). We note that these are examples and the final approach will be determined based on the distribution of the data.

The primary analysis will use an intent-to-treat approach with the use of *i.ARTs* as a 1-time exposure, with α=.05, and 2-sided tests. The number of moderators included in the regression model will be used to make a Bonferroni adjustment to the α value. A secondary efficacy analysis will explore actual treatment taken. Our exploratory tertiary outcomes of viral suppression, clinic attendance, chosen ART modality, and medication refills will compare both between and within *i.ARTs* and control arm women (ie, baseline and postvisit surveys and EMR data at 3 and 6 months); we will also examine these outcomes for women who do not change regimens. We will use prebaseline EMR data to control for previous viral load. We will also compare how many women in each arm change modalities postbaseline to assess *i.ARTs’* accuracy in identifying which HIV treatment modality is the best match. Sensitivity analyses will determine whether assumptions associated with each model are defensible.

#### Power Analysis and Sample Size

Our sample size is based on our secondary outcome of decisional conflict, as our primary outcomes of acceptability and feasibility are only assessed in the intervention group (as the control group will not experience *i.ARTs*). Scale developers use a moderate effect size (0.3-0.4) to determine sample size. Using the average weighted means and SDs from prior studies [52], we calculated sample size for α=.05, power=0.8 for an independent samples *t* test. Assuming a mean score of 21 (SD 16) in the intervention (*i.ARTs*) group and a mean score of 28 (SD 18) in the control group, the sample size of 90 in each group, for a total of 180, provides 80% power. Our tertiary outcomes are exploratory, but as 35% of US women are not adherent after 6 months, we are powered to detect basic differences between the intervention and control arms.

### Ethics Approval

This study received approval from the Columbia University Institutional Review Board (IRB; approval number IRB-AAAT5314), and we will receive approval from University of Miami IRB once the clinical trial portion begins.

## Results

This study was funded in March 2021. Protocols for phase 1 interviews have been developed and interviews with service providers started in September 2021. We will apply for Clinicaltrials.gov registration prior to phase 3, which is when our first participant will be enrolled in the randomized controlled trial. This is anticipated to occur in April 2023.

## Discussion

### Research Implications

This paper describes the protocol for developing and piloting the first decision aid to facilitate women’s decision making between LAI and oral ART modality for HIV treatment. This study will expand HIV treatment research in important ways. The field of HIV care has increasingly emphasized patient-centered care [42], as this approach improves quality of life, patient adherence, and health outcomes [28,42,71]. Patient decision aids are a key tool for patient-centered care, as they can enhance equity in medicine, activate patients to increase their knowledge and decision-making power, and lower medical paternalism [24,29-31]. They also enhance patient care by enabling shared discussions and outcomes that match patients’ needs [26,42,72].

As noted, patient decision aids have been successfully used in other areas of medicine, most similarly to this in facilitating contraception decision making among women [40,41]. However, as yet no patient decision aids exist to facilitate the decision-making process between oral and LAI HIV treatment. Thus, *i.ARTs* is urgently needed to promote equity in patient-provider decision making by helping WWH identify their preferences for oral or LAI ART. Further, developing *i.ARTs* specifically for women may help promote gender equity in the uptake of LAI ART. Examples from oral pre-exposure prophylaxis (PrEP) scale up show that a lack of decision aid tools can hinder uptake and lead to gender-related disparities [73-76]. Women not only have much lower rates of PrEP use than men but also have shorter periods of sustained PrEP use [75,77,78]. Our study to develop and pilot test the *i.ARTs* patient decision aid aims to prevent a similar gender gap for LAI ART uptake and thus a further exacerbation of disparities in care and treatment outcomes.

Furthermore, this study comes at a crucial moment in HIV treatment. As the menu of options for ART treatment expands, patients and providers will encounter different considerations and concerns regarding the various ART modalities. Despite LAI ART’s potential, we know little about the “real-world” facilitators and barriers WWH will face, or how providers will decide to whom to offer LAI ART. The FDA approved LAI ART in January 2021, and developing *i.ARTs* as LAI ART is rolled out has the potential to capture and address some of these real-world barriers and facilitators. In turn, it will provide data that can support the further dissemination of this new biomedical technology. In addition, *i.ARTs* can be updated to incorporate future ART modalities (eg, monthly oral medication [79,80], implants [81], subcutaneous injections [82]).

### Study Strengths

Our study has multiple strengths that ensure the validity and applicability of its findings. As described in the previous sections, we incorporate patient and provider perspectives throughout *i.ARTs* development (phase 1, phase 2) and testing (exit interviews in phase 3). This ensures that *i.ARTs* content includes the full landscape of factors that influence LAI ART uptake, determine whether LAI or oral ART best fits a woman’s values and preferences, and will help us to identify potential differences in patient and providers’ beliefs regarding who should be offered LAI ART.

Next, our real-world clinic-based sample can provide insight into LAI ART uptake that samples of clinical trials participants cannot. As mentioned, women were underrepresented in LAI ART clinical trials [10-13]. Working with women from the MWCCS not only resolves this gender gap, but also provides data that trial- and clinic-based samples (eg, CFAR Network of Integrated Clinical Systems [CNICS] sites) cannot, as it includes out-of-care women who may face different and additional adherence barriers. Clinical trials include participants with high adherence to determine drug efficacy, but only 49% of MWCCS women would be eligible for AIDS Clinical Trials Group trials [44]. Furthermore, pilot testing *i.ARTs* in Miami, which leads the United States in HIV incidence, and whose HIV epidemic is marked by stark racial and ethnic disparities, will help us identify the full landscape of WWH’s barriers to ART use and potential solutions to address them. This will increase the relevance of *i.ARTs* for WWH across the United States.

Finally, our study benefits from its mixed methods approach [83], which increases validity by capitalizing on each method’s inherent strengths. The different methods answer complementary research questions: we will use the phase 1 survey and provider interviews to explore potential disconnects between who may be offered LAI ART and who wants it, which can affect patient equity; interviews with women who choose LAI ART will explore the mechanisms that drive viral suppression data abstracted from EMRs. We will integrate mixed method findings using “triangulation” (ie, use of 2 different methods to address the same research question) to improve reliability [84,85]. Consistency of findings across methodological approaches increases validity and reliability and suggests findings are not due to methodological artifacts [86]. This will allow us to see how themes vary across methods, leading to stronger findings than using each method independently.

### Expected Challenges

As this study is set in real-world clinical settings, we expect challenges to incorporating *i.ARTs* delivery into existing clinical workflows. These include considerations such as available space for participants to complete *i.ARTs*, tech support for the computerized tool and printout, staffing resources, and limited clinician time with each patient to discuss the decision aid results. The study team will work with the clinic staff to determine how to ensure efficient delivery of *i.ARTs* in the clinical setting. Exit interviews will be used to collect data on these challenges to inform further scale-up or dissemination efforts of *i.ARTs* outside of the Miami-based clinic*.*

As LAI ART is being administered in a real-world setting, participating clinicians are subject to existing, and possibly changing, FDA approvals. While phase 3 trials are currently testing LAI ART for nonadherent patients, *the FDA-approved LAI ART formulation is only for virally suppressed patients*. To ensure *i.ARTs* incorporates all relevant drivers of ART use for all women, we aim to include nonadherent women in aim 3, pending FDA approval. If FDA approval only exists for virally suppressed WWH when aim 3 starts, then we will limit the pilot test to that population. Should FDA approval for nonadherent patients begin in the middle of the pilot, we may adjust eligibility criteria to ensure a standard participant population throughout the pilot period. The study team will carefully monitor developments in ART treatment technologies and adapt accordingly. In addition, LAI ART was initially approved as a monthly injection, and the US FDA has approved a bimonthly (ie, every 8 weeks) formulation of LAI ART. This may also affect women’s preferences as the study is rolled out.

New modalities of ART delivery may emerge prior to the pilot study period, including monthly oral medication [79,80], implants [81], and subcutaneous injections [82]. The addition of these new technologies could further complicate the decision-making process. As mentioned earlier, the *i.ARTs* tool is web based and thus easy to update as technology or treatment modalities change [87]. The web-based nature of this tool will allow us to adapt and expand *i.ARTs* to all genders in future studies. In addition, a similar approach could be used to develop a tool to facilitate women’s decision-making process between oral and LAI versions of PrEP, with a goal to limit HIV infections among women.

## Abbreviations

ART: antiretroviral therapy
CNICS: CFAR Network of Integrated Clinical Systems
EMR: electronic medical record
FDA: Food and Drug Administration
*i.ARTs*: i.ART+support
IPDAS: International Patient Decision Aids Standards Collaboration
IRB: institutional review board
LAI ART: long-acting injectable ART
MWCCS: MACS/WIHS Combined Cohort Study
PrEP: pre-exposure prophylaxis
WWH: women with HIV
